# Lumbar postoperative *Aspergillus flavus* infection after lumbar spondylolisthesis fusion: a case report and literature review

**DOI:** 10.3389/fmed.2026.1879141

**Published:** 2026-07-22

**Authors:** Honghai Wang, Xiaojun Han, Hui Zeng, Bo Liu, Cheng Chen, Gangqiang Wu

**Affiliations:** Department of Orthopedics, The Second People's Hospital of Fuyang City, Fuyang, China

**Keywords:** *Aspergillus flavus*, lumbar spondylolisthesis, metagenomic next-generation sequencing, postoperative infection, spinal fusion surgery

## Abstract

Surgical site infection following lumbar internal fixation and fusion is predominantly bacterial. Aflatoxin-associated discitis is extremely rare in immunocompetent patients and often results in delayed diagnosis and inadequate empirical antimicrobial treatment. This report presents a 74-year-old immunocompetent male patient who underwent elective posterior lumbar interbody fusion for grade II degenerative lumbar spondylolisthesis and developed intractable low back pain 3 months postoperatively. Despite multiple courses of broad-spectrum antibiotic therapy administered at two external hospitals, his symptoms did not resolve. Conventional bacterial, mycobacterial, and fungal cultures, as well as histopathological examination of percutaneous biopsy and intraoperative specimens, yielded negative microbial results. Metagenomic next-generation sequencing (mNGS) specifically identified *Aspergillus flavus* in all tissue samples, confirming the etiological diagnosis of fungal discitis. The patient received staged combined antifungal and surgical management. Intravenous voriconazole was used for induction therapy, followed by radical debridement of infected spinal tissue, internal fixation revision, and bone graft reconstruction. Oral voriconazole was prescribed for 3 months of postoperative maintenance therapy. A 12-month follow-up showed marked pain relief, and serial imaging and laboratory tests confirmed complete eradication of the infection with no recurrence. This case is systematically compared with previously reported *Aspergillus* spinal infections in immunocompetent hosts. mNGS serves as a valuable adjunctive diagnostic tool for clinically suspected atypical infections when conventional examinations are negative. Although limited by a single-case, single-center design without statistical generalizability, this report expands clinical recognition of post-fusion fungal discitis in immunocompetent patients and provides practical evidence for precise diagnosis and individualized management of refractory spinal surgical site infections.

## Introduction

Lumbar spondylolisthesis is a common degenerative spinal disease. Posterior lumbar interbody fusion (PLIF) is the most widely adopted surgical approach for the treatment of lumbar spondylolisthesis, with mature surgical techniques and favorable clinical outcomes (1–3). PLIF is selected for patients with grade II spondylolisthesis, intervertebral disc degeneration, and spinal instability, as it achieves direct nerve decompression, slip reduction, and circumferential interbody fusion, which constitute the primary surgical indication of the index case. Postoperative infection is a severe complication of spinal fusion, with an incidence of 1–10% (4, 5). These infections are primarily caused by Gram-positive bacteria, such as *Staphylococcus aureus*, followed by Gram-negative bacteria, with fungal pathogens being rare. *Aspergillus flavus* is an opportunistic fungus that mainly affects immunocompromised patients with chronic illnesses, malignancies, or prolonged exposure to immunosuppressants and glucocorticoids, with extremely rare spinal infections reported in immunocompetent individuals. Postoperative lumbar fungal infection manifests non-specifically, primarily as persistent lumbago and local tenderness with minimal fever. Such ambiguous symptoms are frequently misdiagnosed as bacterial or non-infectious postoperative pain, leading to diagnostic delay (6). Negative results from routine microbiological cultures and pathological tests further hindered early diagnosis. As a novel detection tool, mNGS enables rapid, culture-free, broad-spectrum pathogen identification, offering distinct strengths for detecting rare fungal infections.

Notably, mNGS is not recommended as an initial routine diagnostic test for suspected spinal infection under current clinical management standards. Conventional bacterial culture, acid-fast bacilli screening, inflammatory biomarker testing, and imaging should be performed first. mNGS serves as a complementary diagnostic modality reserved for refractory cases with persistent clinical suspicion of infection despite negative routine microbiological results. In recent years, it has demonstrated unique superiority in diagnosing rare and refractory infections. This article reports a case of *Aspergillus flavus* infection of the lumbar spine after lumbar spondylolisthesis fusion in an immunocompetent patient. After multiple rounds of empirical antimicrobial treatment failed, the patient was finally diagnosed via mNGS and was cured after antifungal therapy combined with reoperation. This case not only expands the clinical spectrum of rare spinal infections but also provides essential insights for the diagnosis and treatment of refractory postoperative lumbar infections.

## Case report

The patient was a 74-year-old immunocompetent male with no chronic comorbidities or classic infectious risk factors, including hypertension, diabetes, coronary artery disease, long-term steroid or immunosuppressant administration, smoking, and alcohol abuse. Five months before the current admission, he underwent L4/5 posterior lumbar interbody fusion for Meyerding grade II degenerative lumbar spondylolisthesis at a local hospital. The initial 7-day hospitalization included standardized 48-h perioperative prophylaxis with second-generation cephalosporins. The surgical incision healed uneventfully with no swelling or exudation at discharge, and no postoperative medications were continued. The patient also denied any history of spinal injection, acupuncture, tuberculosis, or chronic pulmonary disease, eliminating major predisposing conditions for *Aspergillus* colonization.

**Figure 1:**
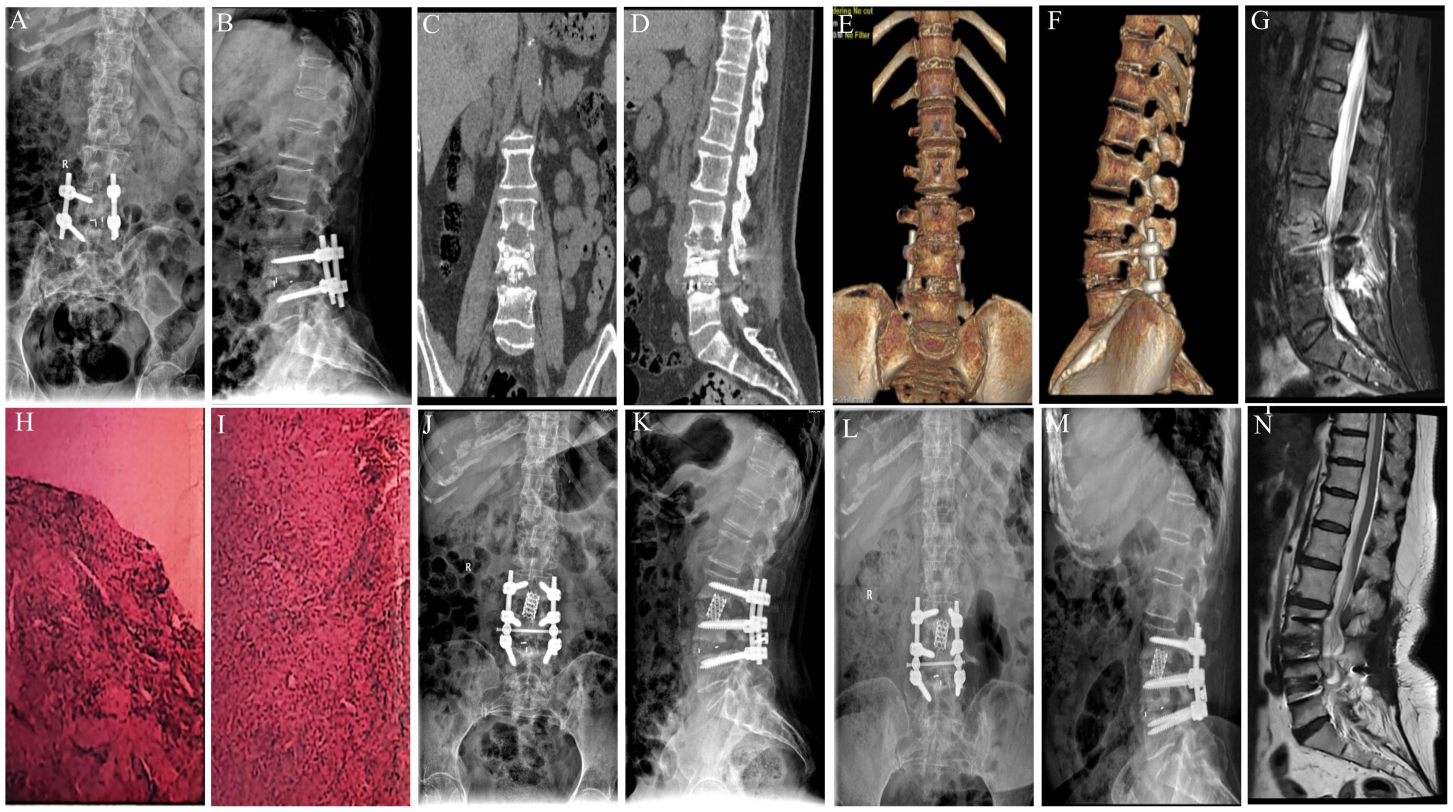
**(A,B)** Anteroposterior and lateral radiographs of the L4–L5 vertebral body fusion surgery. **(C–F)** A lumbar CT plain scan with three-dimensional reconstruction following L4–L5 vertebral body fusion surgery, which suggests bone destruction of the L3-L5 vertebrae, with soft tissue swelling consistent with infection. **(G)** The enhanced MRI T2WI sequence of the lumbar spine after L4-L5 vertebral fusion surgery, which indicates the postoperative changes of the L4 and L5 vertebral bodies and the abnormal signals of the L3-L5 vertebral bodies and intervertebral discs, considering the postoperative infection of the lumbar spine. **(H,I)** The pathological findings of L3-L5 vertebral lesion tissue, which showed inflammatory granulation tissue, necrotic tissue, fibrous tissue, and fibrocartilage tissue, with numerous chronic inflammatory cell infiltrates. **(J,K)** The anteroposterior and lateral X-ray films at 1 month after lumbar lesion clearance and lumbar interbody refusion. **(L,M)** The anteroposterior and lateral lumbar X-ray films at 4 months after lumbar lesion clearance and lumbar interbody refusion. **(N)** The lumbar MRI T2WI sequence at 6 months after lumbar lesion clearance and lumbar interbody refusion, which indicates that the signal intensity of L3-L5 intervertebral discs was reduced to varying degrees, the internal fixation device was in place, the interbody fusion was good, and the vertebral body was not damaged.

Three months after lumbar fusion, the patient developed unexplained low back pain accompanied by tenderness at the surgical incision, without fever, lower extremity numbness or weakness, or bladder and bowel dysfunction. The patient presented to a county hospital 4 days after the onset of pain. At that time, because of limited intervertebral disc puncture skills at the presenting hospital, targeted percutaneous lesion biopsy was not performed to identify the pathogen, so empirical second-generation cephalosporins were initiated for 2 weeks, with no clinical improvement. The symptom gradually worsened and even impaired his daily activities, after which the patient was transferred to a provincial-level hospital for further management. Contrast-enhanced lumbar magnetic resonance imaging (MRI) of the lumbar spine was performed on the 14th day after onset, revealing abnormal soft-tissue signals around the L3-L5 vertebral bodies and adnexa, suggesting a deep surgical site infection (Figure 1). All relevant examinations, including T-SPOT, five ANCA tests, ANA profile, blood culture, and urine culture, returned negative results. One week of empirical vancomycin therapy did not result in symptomatic improvement. After vancomycin was switched to linezolid, the patient developed a rash 1 day after administration, and linezolid was discontinued. Vancomycin was reintroduced, and the rash recurred after 2 days of treatment. The regimen was then adjusted to teicoplanin, which still failed to improve low back pain after 1 week of treatment, with the symptom worsening during turning over and walking. For further diagnosis and treatment, the patient was transferred to our hospital.

Physical examination on admission: body temperature 36.6 °C, heart rate 78 beats/min, respiratory rate 19 breaths/min, and blood pressure 131/78 mmHg; positive tenderness and percussion pain at the L3-L5 lumbar segments; no obvious redness, swelling, or exudation at the surgical incision; normal muscle strength and sensation in both lower extremities; and negative pathological reflexes. Pulmonary-derived *Aspergillus* infection was systematically excluded on admission. Normal chest CT findings, including the absence of fungal-specific nodules, cavities, or infiltrative lesions, combined with the lack of respiratory symptoms, obviated the need for bronchoalveolar lavage and effectively ruled out hematogenous dissemination from a primary pulmonary focus. Laboratory examinations: white blood cell count 4.29 × 10^9^/L, neutrophil percentage 47.2%, C-reactive protein (CRP) 16.1 mg/L, serum amyloid A (SAA) 32.7 mg/L, and erythrocyte sedimentation rate 81 mm/h; no significant abnormalities were observed in routine blood tests, liver and renal function, or electrolyte levels (Table 1). Three-dimensional reconstructed lumbar CT showed postoperative alterations, including bone destruction of the L3-L5 vertebral bodies, accompanied by surrounding soft-tissue swelling, consistent with infection (Figure 1).

**Table 1:** Dynamic changes of main inflammatory indicators during hospitalization and follow-up.

Timepoint	White blood cell count (WBC) (3.5–9.5*10^9/L)	Neutrophil percentage (N%) (40–75%)	Neutrophil percentage (N%) (20–50%)	Monocyte percentage (M%) (3–10%)	Eosinophil percentage (E%) (0.4–8.0%)	Basophil percentage (B%) (0–1%)	C-reactive protein (CRP) (0-6 mg/L)
Preoperative period	4.29	47.2	41.6	7.7	2.4	1.1	16.1
1 day after the operation	14.41	88	4.2	7.7	0	0.1	49.3
3 days after the operation	10.98	76.6	13.2	9.3	0.6	0.3	304.4
5 days after the operation	7.23	63	23.1	9.7	3.8	0.4	190.3
1 week after the operation	9.14	71.9	16.4	9.7	1.6	0.4	191.7
2 weeks after the operation	9.45	68.6	25	5.3	0.8	0.3	13.5
1 month after the operation	3.46	55.8	31	7.9	4.1	1.2	1.2
3 months after the operation	4.88	61.4	30.4	6.1	1.6	0.5	1.8
6 months after the operation	5.90	64.3	23.8	9.8	1.7	0.4	9.1

To rapidly identify the nature of the lumbar infection, a CT-guided lumbar puncture was performed, and lesion tissues were collected for bacterial culture, mycobacterial culture, fungal culture, routine histopathology, mNGS, and rapid molecular identification of rifampicin-resistant tuberculosis. Preoperative puncture specimens yielded negative findings in bacterial, mycobacterial, and fungal cultures, tuberculosis molecular detection, and routine histopathological examination. Microscopic examination merely showed nonspecific inflammatory infiltration, with no fungal hyphae identified. Notably, mNGS testing of lesion tissue uniquely detected *Aspergillus flavus*, with a relative abundance of 23.08% and 23 unique reads, while no other pathogens were identified. Integrating the patient’s persistent clinical symptoms, progressive osteolytic lesions on imaging, and elevated inflammatory markers, the definitive diagnosis of postoperative lumbar *Aspergillus flavus* infection was established based on positive mNGS findings and a favorable response to targeted antifungal treatment.

The full antifungal regimen (drug selection, loading dose, maintenance dose, and total treatment duration) was co-designed by orthopedic and infectious disease specialists, in accordance with the 2024 ESCMID aspergillosis clinical guidelines. After diagnosis, oral voriconazole antifungal therapy was administered. Voriconazole was initially administered at a dose of 400 mg intravenously for 3 days, followed by 200 mg intravenously every 12 h for 4 days. One week after medication, the patient’s low back pain was significantly relieved, with partial declines in inflammatory markers, as shown in Table 1. Considering the patient’s severe vertebral bone destruction, risk of spinal instability, and inflammatory encapsulation around the L4/5 internal fixation, L3-L5 posterior debridement, internal fixation revision, and autologous bone graft fusion were performed 1 week after antifungal therapy (Figure 1). Detailed surgical procedure: (1) Posterior midline incision to expose the posterior structures of L3–L5; (2) Removal of the L4-L5 pedicle screws, rods, and fusion cage; (3) Complete removal of the disc spaces at L3-L4 and L4-L5, along with surrounding pathological tissue; (4) Placement of two appropriately sized pedicle screws into each of the L3, L4, and L5 vertebral bodies; (5) Insertion of a 12 mm diameter titanium mesh through the right intervertebral foramen at L3/4, and placement of a 26 × 10 × 14 mm fusion cage through the right intervertebral foramen at L4/5; (6) Replacement with a longer connecting rod to stabilize and fix the L3-L5 vertebrae. At 4 months postoperatively, follow-up X-rays showed mild subsidence of the titanium mesh, which was clinically insignificant, without axial low back pain or radicular symptoms; thus, no further intervention was required. Surgical lesion samples were negative for routine bacterial, mycobacterial, and fungal cultures as well as histopathological staining, with no fungal hyphae seen microscopically; only lesion mNGS was positive for *Aspergillus flavus*. After the operation, the patient continued to take voriconazole for antifungal therapy for 3 months, and no obvious abnormality was found in liver and kidney function during regular reexamination throughout the medication period.

At the 1-month postoperative follow-up, the patient’s spontaneous and activity-induced back pain had substantially subsided; inflammatory markers were markedly improved (Table 1). At 6 months postoperatively, lumbar MRI reexamination showed no abnormal signal in the L3-L5 vertebral bodies or intervertebral discs, with preliminary bony bridging around the fusion cage (Figure 1). Postoperative interbody fusion was graded as Bridwell II at 6 months using a standardized classification system. Serial CT surveillance was not performed owing to the patient’s reluctance to undergo repeated radiation exposure; therefore, MRI was adopted for postoperative monitoring to exclude residual epidural and paraspinal soft-tissue inflammation, a key indicator of fungal discitis. At the 12-month final follow-up, the patient achieved complete relief of low back pain, with the visual analog scale score improved from 7/10 preoperatively to 0/10. Full functional recovery was attained, allowing unrestricted daily activities and light physical work. No infection recurrence, neurological deficits, or progressive vertebral collapse was identified during long-term follow-up.

Before receiving targeted antifungal therapy and surgical treatment, the patient reported severe sleep disturbances and was unable to stand or walk for more than 5 minutes. After staged interventions, the patient regained full self-care ability and expressed satisfaction with the long-term clinical outcome, with no ongoing discomfort.

**Figure 2:**
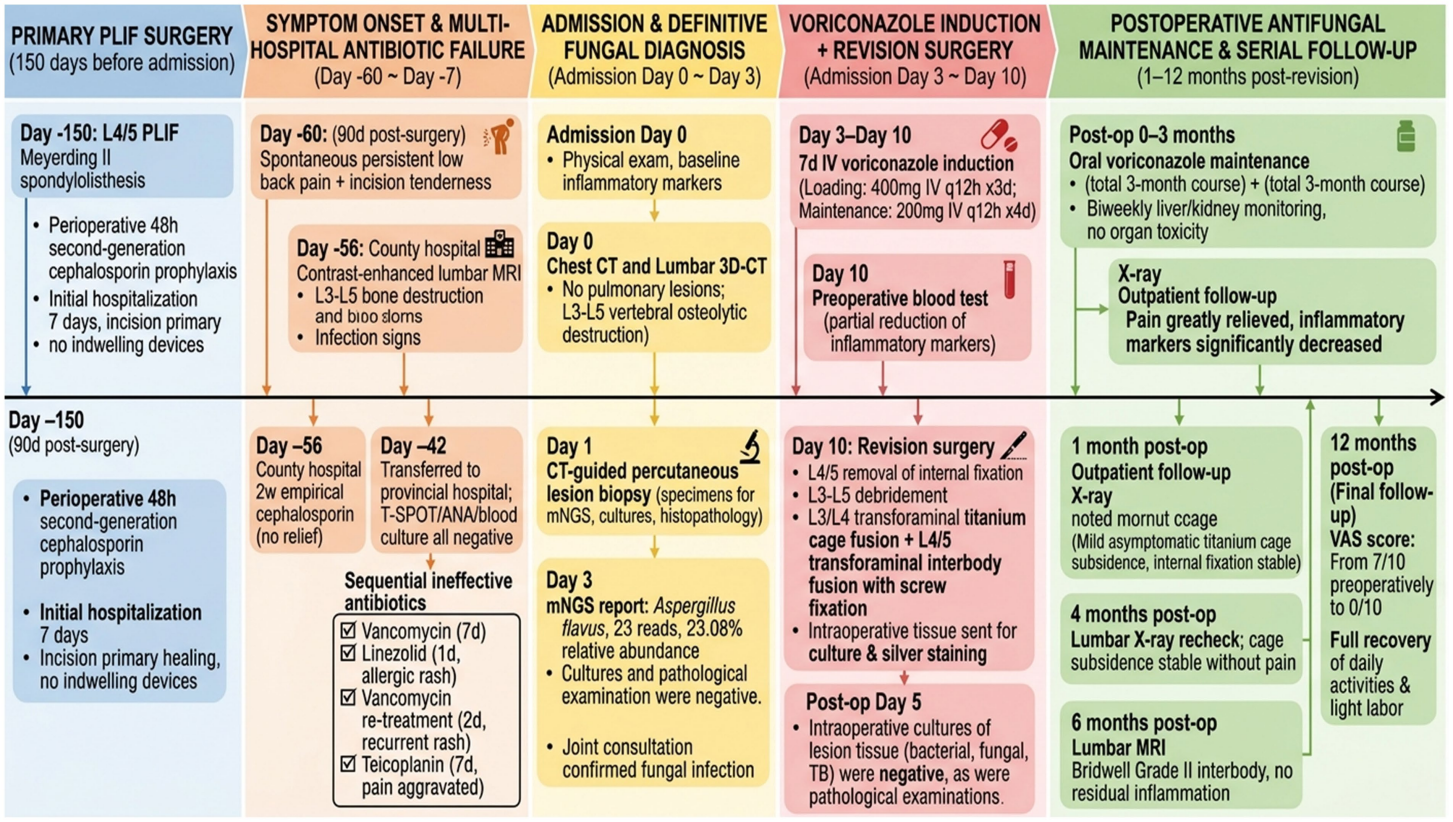
Clinical timeline showing the progression of disease, diagnostic procedures, treatment regimens, and follow-up outcomes of the patient.

To intuitively summarize the entire clinical pathway, we constructed a clinical timeline covering disease progression, diagnosis, treatment, and follow-up (Figure 2).

## Discussion

Surgical site infection is a severe complication of instrumented lumbar fusion, with invasive postoperative spinal aspergillosis occurring extremely rarely and primarily in immunocompromised patients. As a widespread environmental fungus, *Aspergillus* rarely causes disease in immunocompetent individuals, and the clinical profiles of these rare spinal infections remain poorly characterized. Addressing this research gap, this systematic review analyzed relevant literature published between 2015 and 2025 from PubMed, Web of Science, and CNKI, focusing on *Aspergillus* spondylodiscitis and postoperative spinal fungal infection in immunocompetent adults. The search strategy employed terms covering immunocompetent status, *Aspergillus* spondylodiscitis, spinal fungal infection, and postoperative spinal infection. Rigorous inclusion and exclusion criteria were applied to screen eligible cases: only adult patients with histologically or microbiologically confirmed *Aspergillus* vertebral infection and no underlying immunosuppressive disorders were included, whereas those with diabetes mellitus, long-term steroid administration, malignancy, organ transplantation, or HIV infection were excluded. Multiple standardized clinical indicators were extracted and analyzed from all qualified cases, including predisposing risk factors, clinical presentations, imaging features, diagnostic methods, pathogenic *Aspergillus* species, combined medical and surgical treatment regimens, total antifungal treatment duration, follow-up period, disease-related complications, and ultimate clinical prognoses. All cases that met the unified screening criteria were finally consolidated and summarized in Table 2.

**Table 2:** Clinical characteristics of *Aspergillus* spondylitis in immunocompetent patients.

Year/Reference	Sex/Age	Predisposing factors	Clinical manifestations	Radiological features	Diagnostic approach	Pathogen species	Treatment regimen	Outcome
2015/(7)	M/20	Traumatic inoculation, previous pulmonary tuberculosis	Cough, fever, back pain	T11 fracture, paravertebral abscess at the T7 level	CT-guided spinal biopsy + fungal culture	*Aspergillus terreus*	Voriconazole 16 days + caspofungin 40 days	Sequelae
2015/(8)	M/53	Pulmonary mass, multiple pulmonary nodules	Cough, fever, low back pain	L2-L5 vertebral body bone destruction, no internal fixation	Surgical specimen histopathology	*Aspergillus* species	Debridement + voriconazole 3 months	Recovered
2019/(9)	M/61	Lumbar steroid injection	Mild low back pain	L3-L4 vertebral bodies destruction, kyphosis	*Intervertebral disc space biopsy +* fungal culture	*Aspergillus nidulans*	Laminectomy+fusion+caspofungin 6 weeks+ voriconazole 7 months	Recovered
2019/(10)	M/71	Abdominal stab wound	Back pain, leg numbness	T11 and T12 vertebral bodies and destruction of the endplate of T11-T12	Surgical specimen histopathology + blood examination.	*Aspergillus terreus*	Laminectomy+fusion+ voriconazole 5 months	Recovered
2020/(11)	M/43	Brucellosis-related vertebral osteo-myelitis	Low back pain, leg pain	L5-S1 spondylodiscitis with an epidural abscess	Immunohistochemistry + fungal culture	*Aspergillus* species	Voriconazole 12 weeks	Recovered
2022/(12)	F/58	None	Low back pain, fever	T11–T12 intraspinal abscess with discitis	Surgical specimen histopathology	*Aspergillus* species	Laminectomy + voriconazole 3 months	Recovered
2023/(13)	M/68	None	Thoracolumbar back pain	Destruction of T12-L1 and L2 with vertebral collapse	Surgical specimen histopathology + fungal culture	*Aspergillus fumigatus*	Reconstructive spinal surgery+ voriconazole 2 months+ isavuconazole 10 months	Recovered
2023/(14)	F/58	Acupuncture and acupotomy	Low back pain	Destruction of the L4-L5 vertebral bodies	Surgical specimen histopathology + fungal culture	*Aspergillus terreus*	Laminectomy+fusion + voriconazole 6 months	Recovered
2024/(15)	M/63	None	Low back pain, limb pain and numbness	Destruction of the T12-L1 vertebral bodies	Surgical specimen histopathology + fungal culture	*Aspergillus fumigatus*	Laminectomy+fusion + voriconazole 1 month	Recovered
2025/(16)	M/27	None	Back pain	Destruction of the L3-L4 vertebral bodies	Surgical specimen histopathology+mNGS	*Aspergillus fumigatus*	Surgical debridement and internal fixation+ voriconazole 10 weeks	Recovered
2025/Our case	F/71	Lumbar fusion	Back pain	L3-L5 osteomyelitis, no paravertebral abscess, L4/5 internal fixation on intact	Surgical specimen histopathology+mNGS	*Aspergillus flavus*	Lumbar lesion radical debridement+refusion + voriconazole 3 months	Recovered

The average age of these patients was 53.9 years; 72.7% were male, and the most common clinical manifestation was back pain, occasionally accompanied by fever or neurological dysfunction. Lumbar involvement was the most common, accounting for 63.6% of cases, and the overall treatment outcome was good, with 90.9% of patients recovering completely; however, one patient developed severe sequelae. Among the 11 cases, 81.8% of patients received a combination of antifungal therapy and surgical intervention, and treatment duration ranged from 1 month to 12 months (7–16). Unlike the other 10 cases, our case involved an aflatoxin infection after lumbar fusion in an immunocompetent patient without underlying disease, which is an incidental event in clinical practice and a highlight of this case. The main causes for the difficulty in early diagnosis and misdiagnosis of this case are as follows: first, the patient only presented with lower back pain and incision tenderness without fever, which differs from typical bacterial spinal infections (often accompanied by fever and elevated inflammatory indicators) and spinal tuberculosis (often accompanied by low-grade fever, night sweats, and weight loss); second, the results of conventional bacterial culture, tuberculosis-related examinations (T-SPOT), and autoimmune-related examinations were all negative, preventing the timely identification of the pathogenic bacteria; third, empirical antibacterial therapy failed to achieve efficacy, but the possibility of fungal infection was not considered promptly. The patient did not respond to treatment with multiple broad-spectrum antibiotics (second-generation cephalosporins, vancomycin, teicoplanin). Furthermore, the attending physician did not recognize that this was a rare fungal infection, leading to a delayed diagnosis.

In recent years, metagenomic next-generation sequencing (mNGS) has been widely used to diagnose rare, refractory, and unknown pathogens. Its core advantage lies in enabling direct detection of pathogenic microbial nucleic acids in clinical samples without prior culture, featuring rapid detection, broad coverage, and high sensitivity (17–19). For rare fungal infections such as *Aspergillus flavus*, traditional fungal culture has drawbacks, including a long cycle (3 ~ 7 days) and a low positive rate. In contrast, mNGS can detect pathogenic bacteria within a short period (24 ~ 48 h), providing critical evidence for early diagnosis and targeted therapy. In this case, mNGS detected *Aspergillus flavus* in the lesion tissue, with a relative abundance of 23.08% and 23 reads detected in total. No other pathogenic bacteria were identified, consistent with the patient’s response to clinical antifungal therapy and confirming the reliability of mNGS results. This case indicates that, for refractory post-spinal surgery infections that do not respond to multiple antimicrobial treatments and test negative on routine examinations, mNGS should be performed as early as possible to identify the pathogenic bacteria and avoid delayed or missed diagnosis. This conclusion is of great practical significance for clinical spinal surgeons and also represents the core innovation of this case report. Despite its diagnostic value, mNGS still has notable limitations in identifying spinal aspergillosis. Existing relevant evidence is confined to sporadic case reports, with no large-cohort studies validating its diagnostic efficacy, which precludes robust statistical evaluation of its overall sensitivity and specificity for this infection. In the present case, repeated conventional microbiological and histopathological examinations of both preoperative biopsy specimens and intraoperative lesion tissues returned negative results. By contrast, mNGS successfully identified *Aspergillus flavus* as the causative pathogen, and the favorable clinical response to subsequent voriconazole monotherapy further validated the accuracy of the detection. For postoperative refractory spinal infections that remain unconfirmed by routine culture and histopathology and fail to respond to empirical antibiotic regimens, timely mNGS detection is highly recommended to facilitate the identification of rare fungal pathogens and reduce missed diagnoses.

To clarify the pathogenic mechanism of spinal aspergillosis in this immunocompetent patient, we analyzed two plausible routes of infection: hematogenous dissemination and intraoperative direct inoculation. The absence of symptoms and negative chest CT findings ruled out a primary pulmonary *Aspergillus* focus, excluding hematogenous seeding as a valid etiology. Furthermore, the patient reported no cutaneous trauma, intravenous catheterization, or extra-spinal invasive procedures that could precipitate fungemia. Given that the infectious lesion was strictly restricted to the initial PLIF surgical field, direct intraoperative inoculation of *Aspergillus flavus* spores during spinal instrumentation is proposed as the predominant infection pathway. Potential contributing factors may include intraoperative exposure to environmental mold, contaminated internal fixation devices, or surgical irrigation solutions. However, the precise source of contamination cannot be fully confirmed through retrospective analysis, which constitutes an unavoidable limitation of the present single-case study.

Standardized treatment guidelines are lacking for instrumented lumbar *Aspergillus flavus* spondylodiscitis, whose management currently relies on international criteria and clinical experience, combining long-term targeted antifungal therapy and radical surgical debridement for vertebral destruction or implant-related fungal colonization. As recommended by ESCMID guidelines, voriconazole is the first-line agent for invasive aspergillosis (20), with potent anti-Aspergillus activity and satisfactory osseous and paravertebral tissue penetration. Guided by infectious disease consultation and summarized clinical evidence (Table 2), this patient received a 12-week voriconazole regimen, a proven-effective course to eradicate residual fungal colonization and prevent postoperative recurrence; routine liver and renal function monitoring was conducted throughout treatment without adverse toxic effects. Given the patient’s severe systemic inflammation, staged treatment was adopted to avoid the risks of primary debridement, including inflammatory exacerbation, massive hemorrhage, and fungal dissemination. Preoperative voriconazole induction inhibited fungal activity and alleviated local inflammatory edema, while standardized intraoperative irrigation and abscess resection effectively prevented neurological complications. Revision surgery was performed due to destabilizing L3–L5 osteolytic lesions and retained pedicle screws that formed avascular niches hindering antifungal penetration. The transforaminal approach minimized surgical trauma, and follow-up imaging revealed mild, asymptomatic cage subsidence that required no secondary intervention. Fusion was assessed using the Bridwell grading system, and MRI was preferred for postoperative surveillance in place of repeated CT scans to avoid cumulative radiation exposure, with residual soft-tissue inflammation as the primary therapeutic evaluation index.

This case delivers a practical clinical reference for the diagnosis and treatment of postoperative fungal spondylodiscitis. Clinically, it reminds clinicians to consider rare *Aspergillus* infection in antibiotic-refractory postoperative spinal infections, even in immunocompetent patients without conventional immunosuppressive risk factors. It also validates the diagnostic value of mNGS combined with comprehensive microbiological detection and confirms the safety and efficacy of standardized staged voriconazole therapy plus individualized debridement and reconstruction for delayed instrumented *Aspergillus flavus* spondylodiscitis. Several limitations exist in this study. As a single-case observation lacking cohort control, its findings cannot be broadly generalized. The exact intraoperative contamination source is untraceable, and the underlying infection mechanism remains presumptive. Postoperative serial CT fusion evaluation was abandoned due to patient radiation concerns, limiting quantitative assessment of long-term bone graft fusion. Furthermore, current studies are insufficient to determine the true diagnostic accuracy of mNGS for spinal aspergillosis, and further large-scale prospective verification is needed.

## Conclusion

This study describes a rare case of delayed *Aspergillus flavus* spondylodiscitis following lumbar fusion surgery in an elderly immunocompetent patient. Diagnostic delay was largely attributable to atypical clinical manifestations and consistently negative microbiological and histopathological findings from both preoperative puncture samples and intraoperative lesion tissues. Pathogenic identification was finally achieved through integrated CT-guided mNGS, intraoperative fungal culture, and silver staining. The patient obtained a favorable recovery after staged voriconazole antifungal treatment combined with surgical debridement and spinal reconstruction. This case highlights the uncommon nature of postoperative *Aspergillus* spinal infection in immunocompetent recipients of instrumented spinal surgery. It further supports mNGS as a valuable auxiliary diagnostic method for refractory, culture-negative spinal infections, rather than a routine first-line test, and verifies the effectiveness of personalized multidisciplinary therapeutic regimens. This work enriches the clinical data concerning rare postoperative fungal spondylodiscitis and provides practical references for orthopedic practitioners. For intractable postoperative spinal infections that fail empirical antibiotic therapy and yield negative conventional laboratory results, early mNGS testing can help detect rare fungal pathogens, enabling targeted antifungal intervention to minimize complications and optimize long-term prognosis.

## Data Availability

The original contributions presented in the study are included in the article/supplementary material, further inquiries can be directed to the corresponding author.
